# Permeability-Enhanced Liposomal Emulgel Formulation of 5-Fluorouracil for the Treatment of Skin Cancer

**DOI:** 10.3390/gels9030209

**Published:** 2023-03-09

**Authors:** Ankur Pachauri, Havagiray Chitme, Sharad Visht, Vijay Chidrawar, Nawaj Mohammed, Basel A. Abdel-Wahab, Masood Medleri Khateeb, Mohammed Shafiuddin Habeeb, Mohamed A. A. Orabi, Marwa B. Bakir

**Affiliations:** 1 Faculty of Pharmacy, DIT University, Dehradun 248009, Uttarakhand, India; 2 Raghavendra Institute of Pharmaceutical Education and Research, Chiyyedu 515721, Andhra Pradesh, India; 3 Department of Pharmacology, College of Pharmacy, Najran University, Najran P.O. Box 1988, Saudi Arabia; 4 Department of Pharmacognosy, College of Pharmacy, Najran University, Najran P.O. Box 1988, Saudi Arabia; 5 Department of Pharmacology, College of Medicine, Najran University, Najran P.O. Box 1988, Saudi Arabia

**Keywords:** 5-fluorouracil, skin cancer, liposomal emulgel, cytotoxicity, clove oil, eucalyptus oil

## Abstract

The plain 5-fluorouracil (5FU) formulations available in the market are associated with adverse effects such as skin irritation, pruritus, redness, blisters, allergy, and dryness on the site of application. The objective of the present study was to develop a liposomal emulgel of 5FU with increased skin permeability and efficacy using clove oil and eucalyptus oil along with pharmaceutically acceptable carriers, excipients, stabilizers, binders, and additives. A series of seven formulations were developed and evaluated for their entrapment efficiency, in vitro release profile, and cumulative drug release profile. The compatibility of drugs and excipients, as confirmed by FTIR (fourier-transform infrared spectroscopy) and DSC (differential scanning calorimetry) as well as SEM (scanning electron microscopy) and TEM (transmission electron microscopy) studies, revealed that the size and shape of liposomes are smooth and spherical, and the liposomes are non-aggregated. To understand their efficacy, the optimized formulations were evaluated for cytotoxicity using B16-F10 mouse skin melanoma cells. The eucalyptus oil and clove oil-containing preparation significantly produced a cytotoxic effect against a melanoma cell line. The addition of clove oil and eucalyptus oil increased the efficacy of the formulation by improving skin permeability and reducing the dose required for the anti-skin cancer activity.

## 1. Introduction

Melanoma is one of the most serious conditions and the 19th most common cancer globally [1]. It arises on any part of the skin, especially the part exposed to the sun [2]. It develops in the melanocytes, the cells known to produce the melanin pigment that gives color to the skin. Similarly, actinic keratosis is one of the most common conditions that arise on the part of the skin exposed to the sun. It is considered an early squamous cell carcinoma and further progresses to invasive squamous cell carcinoma [3,4].

An extensive literature review shows that there are several efforts in place to develop a suitable formulation of 5-fluorouracil (5FU) to make it safer and more convenient for patient use [5]. Micro-sponges of the drug were also prepared to be delivered topically, and subconjunctival injections in stent implantation are seen to be effective in the postoperative management [6]. A microemulsion preparation of 5FU was prepared for parenteral administration. Carbopol-based transfersomal gel of 5FU for skin cancer treatment is found to have better penetration and no irritation [7]. However, its industrial application is questionable, as it is expensive and involves a higher level of technological aspects. Poloxamer-based thermosensitive hydrogels for local delivery of 5FU were prepared for neoadjuvant or adjuvant therapy for colorectal cancer [8]. A modified form of elastic liposome was shown to extract the subcutaneous layer of the skin for better efficacy, and a 5% cream was prepared for actinic keratosis [9,10]. Nanocapsules containing gel, 5FU-NLC gel, and the in-situ gelling enema have proven to be better than the suppository [11]. Carbopol 934-based 5FU bioadhesive gel prepared has its own value for conventional use in clinical aspects [12]. A liposomal crosslinked gel formulation prepared using sodium alginate and hyaluronic acid was prepared for the topical treatment of basal cell carcinoma [13].

Cream and ointment have a number of drawbacks, including a lower spreading coefficient, lower penetration into the stratum corneum, and less patient compliance due to stickiness or the need to rub the product [14,15]. The gels also have the limitation of being unable to distribute hydrophobic medicines. A formulation that reduces the size of vesicles embedded in the gel is necessary, according to a review of the literature, to improve the permeability of the drug through the skin; the oil chosen for the formulation should have a cooling, drying, emollient, or protective effect; good liposomal gel should avoid greasy formulations and acute weepy dermatitis; and the viscosity of the gel must be maintained so that it can be applied to the skin without difficulty; the preparation must behave as a dual control release system; avoid phase separation in gel preparation; and liposomal gel must be transparent, having micro-sized oil droplets without any coalescence [16,17].

Emulgel, a combination of polymer-based gel and emulsion, is advantageous in that it can be used to transport both hydrophobic and hydrophilic medications into the bloodstream, resulting in improved drug release [18]. Emulgels are chosen over other gels and plain emulsions for drug delivery because they are easier to apply and remove, greaseless, thixotropic, emollient, water-soluble, non-staining, and have an extended shelf life [19]. It has been demonstrated that the emulsion component of the emulgel protects the encapsulated drug against hydrolysis and enzymatic degradation without altering the drug’s therapeutic concentration. Where the gel component improves thermodynamic stability by decreasing surface tension and interfacial tension and increasing retention duration and spreadability [20]. If nano-emulsion is prepared and a gelling agent is added, nano-emulgel can be produced. The gelling substance is believed to expand upon liquid absorption. These nanoemulgels have demonstrated efficacy by enhancing bioavailability via a controlled release mechanism, particularly for hydrophobic medicines and pharmaceuticals with a shorter half-life, in addition to possessing a larger loading capacity and causing less discomfort [21]. Any ideal emulgel should be stable, devoid of moisture entrapment, cake formation, oil globule coalescence, and excessive spreadability. These formulations are therefore recommended for topical medication administration [22]. In this experiment, natural eucalyptus oil and clove oil were utilized to enhance biocompatibility and bioavailability. By forming networks with polysaccharides and other biopolymers, they are thought to boost pharmacological activities and improve skin permeability.

The efficacy of a topical medicinal formulation is dependent on the formulation’s ability to permeate the body’s largest protective barrier, the skin. Thus, formulations must be formulated in such a way that the active medicinal component is transported to the site of action by traversing all three layers of skin along with lipids, fats, and water. Due to their compact size, nanoparticles can traverse the stratum corneum via transcellular, intercellular, and transappendageal transport mechanisms [23]. Permeation of emulgel depends on the type of gelling agent, surfactant, and permeation enhancers as well. With the aid of a surfactant or co-surfactant, these substances enhance skin permeability and formulation adhesion when present in the proper proportions [24].

The natural bioactive substances from medicinal plants that have anticancer properties are presently of great interest. For instance, the highly prized spice clove (*Syzygium aromaticum* L.; Family Myrtaceae) has been used traditionally as a food preservative and for a variety of medical purposes [25]. The main active ingredient of *S. aromaticum*, eugenol, has a number of positive qualities, including antioxidant, anti-inflammatory, and anticancer actions [26]. Due to its extensive properties, which include antioxidant, anticancer, anti-inflammatory, and antimicrobial activities, it has been used for a very long time all over the globe. Eugenol’s multidirectional activities continue to attract researchers’ attention because they imply that it could be included in drugs to cure a variety of diseases. Eugenol’s anticancer properties are achieved through a number of pathways, including inducing cell death, cell cycle arrest, inhibiting migration, metastasis, and angiogenesis in a number of cancer cell lines. Eugenol may also be used as an adjunct therapy for individuals receiving conventional chemotherapy [25]. Similarly, the broad range of secondary metabolites found in the essential oil derived from eucalyptus were capable of slowing or inhibiting cancerous cell lines both in vitro and in vivo, demonstrating *Eucalyptus*’ enormous potential to treat malignancies [27].

Given their role in the anticancer action, using this combination of promising Eucalyptus and Clove oils will improve the efficacy of 5FU while lowering its toxicity. Additionally, clove oil and eucalyptus oil have been successfully used in drug delivery formulations like liposomes, nanoliposomes, emulgels, and other related formulations, showing their suitability for use with gelling agents and other formulation ingredients [28].

Taken together, the primary objective of the present study was to develop a liposomal emulgel comprising 5FU and clove oil/eucalyptus oil for the treatment of skin cancer. The prepared liposomal emulgel of crystallized 5FU is anticipated to contain vesicles of nanosize that are stable with an increased duration of release and an improved rate of dissolution and are dispersed throughout to ease the topical application of the emulgel with lower shear stress and cover the required surface area of the skin without any skin irritation [29,30]. Its unique composition will improve retention, effectiveness, penetration, and provide a longer period of action, reducing the frequency of gel application [31,32].

## 2. Results and Discussion

### 2.1. Pre-Formulation Studies

The pH values of all of the prepared formulations ranged from 6.6 to 6.7, which were considered acceptable to avoid the risk of irritation upon application to the topical because the pH of adult skin is 6.5. 5FU was noticed to be a free-flowing, crystalline white powder that had an unpleasant taste and was irritating in its nature. The temperature at which the solid melted was 282 ± 0.51 °C.

### 2.2. Fourier Transform Infrared Spectroscopy (FTIR)

**Figure 1:**
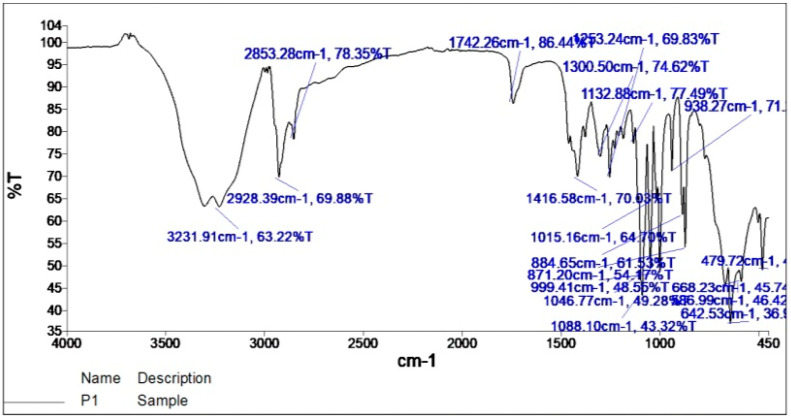
FTIR spectrum of a pure drug.

**Figure 2:**
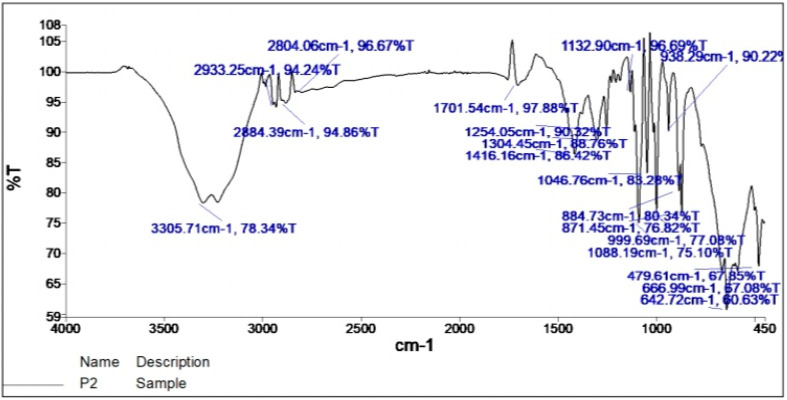
FTIR spectrum of drug-formulated liposomal emulgel.

The IR display was taken by scanning between the infrared absorption frequency range of 4000–400 cm^−1^. The profile and intensity of both FTIR spectra were detected and compared. The FTIR spectra of the pure drug (Figure 1) and the drug-formulated emulgel (Figure 2) evolved peaks in approximately the same range as shown in Figure 1 and Figure 2, indicating the absence of major changes in the functional group [33]. This also proved that the functional groups of drug molecules remained unaltered due to the addition of pharmaceutical excipients and exposure to physical and mechanical stress [34]. The FTIR spectra demonstrate C-H stretching at 2853 cm^−1^. A broad band at 3231 cm^−1^ indicated the stretching of O-H. Further C-O-H bending was noted at 1416 cm^−1^; C-O-C stretching was shown at 1132 cm^−1^; C-O stretching was noted at 1015 cm^−1^; the C=O group was noted at 1742 cm^−1^; the reversal of the band peak at 1701 cm^−1^ in the emulgel indicated the interaction between the carboxylic bond between 5FU and excipients for better stability.

### 2.3. Differential Scanning Calorimetry (DSC)

**Figure 3:**
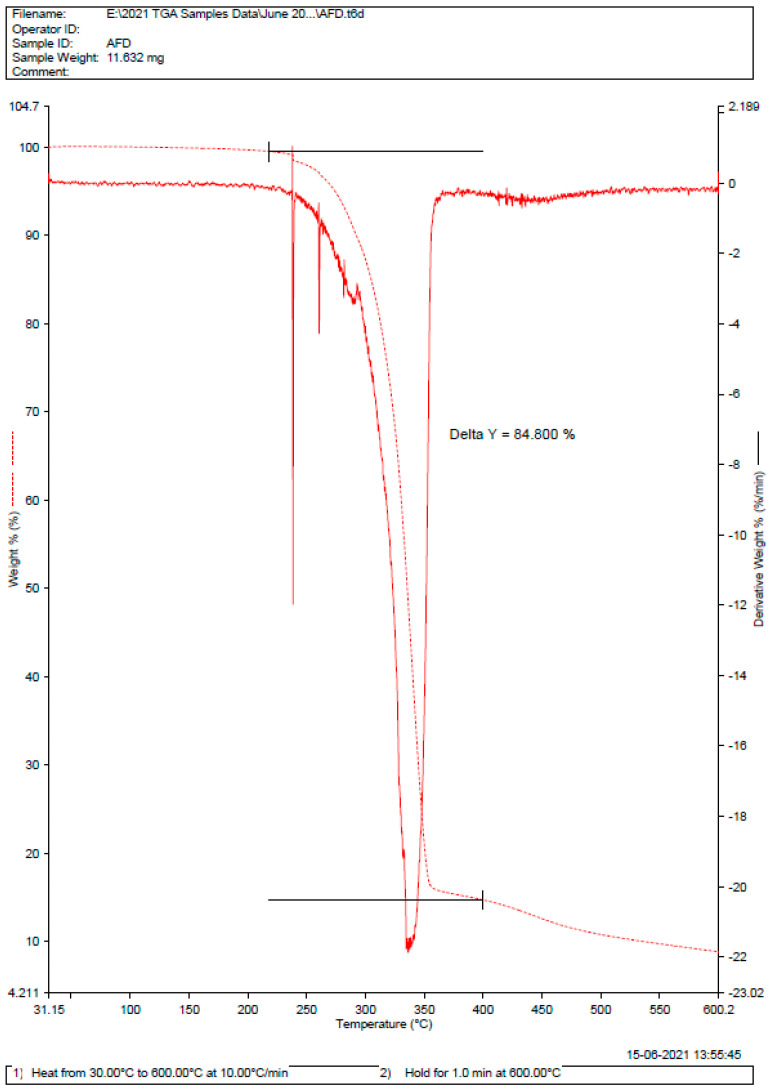
DSC of drug 5FU.

**Figure 4:**
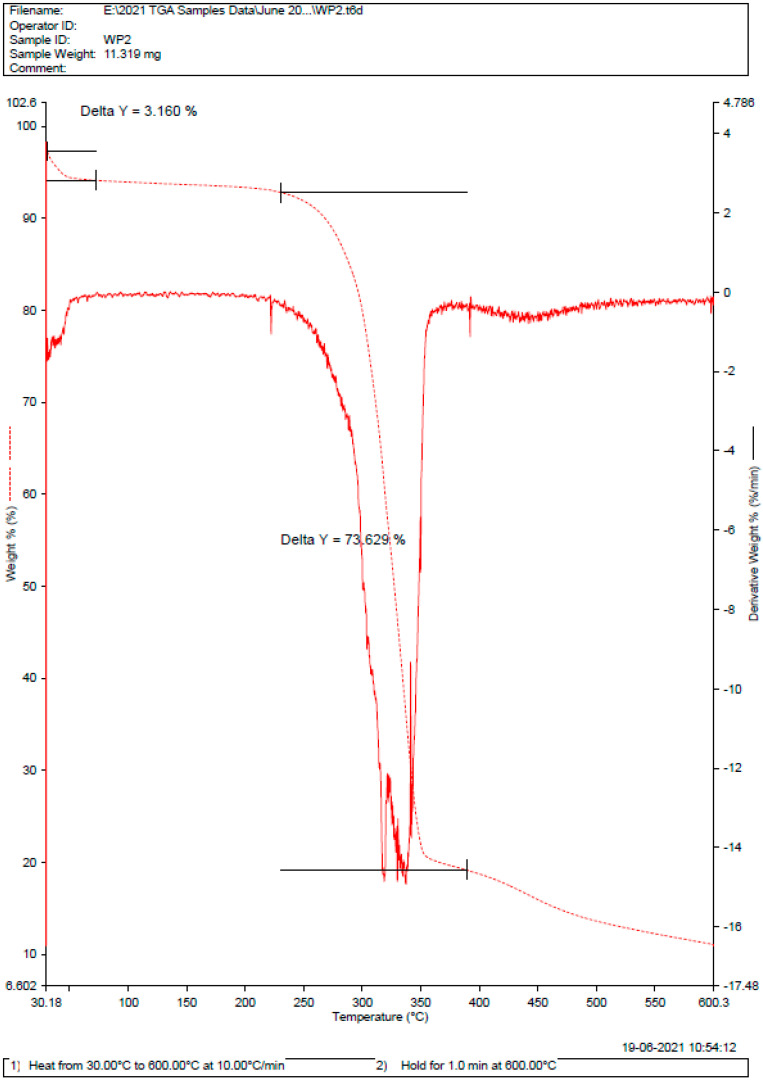
DSC of drug-formulated liposomal emulgel.

As there is no major deviation in the melting point of pure drug and drug in the form of formulation, as shown in Figure 3 and Figure 4. Therefore, there was no incompatibility or instability between a pure drug and a drug-formulated emulgel [35,36]. The DSC curve of 5FU shows a strong curve at 285 °C with a delta of 84.8%. Whereas, for the dispersal of 5FU in emulgel, the delta value was calculated to be 73.629%.

### 2.4. In-Vitro Drug Release Study

**Figure 5:**
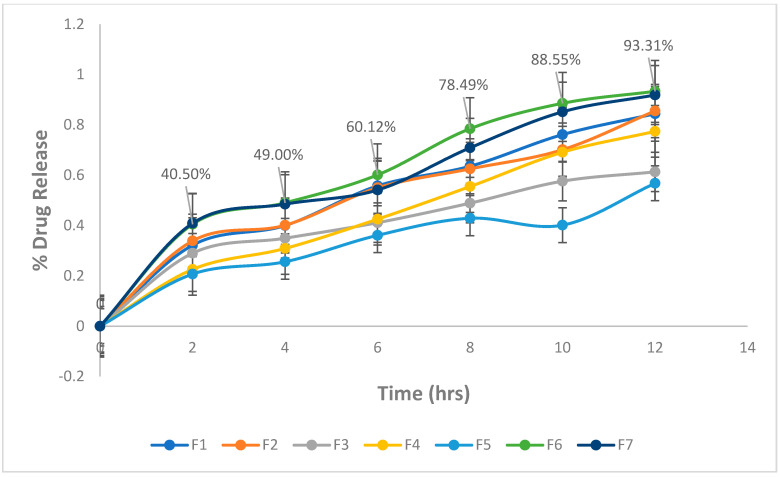
In vitro drug release profiles of formulations.

The in vitro release profiles of 5FU from its various emulgel formulations were observed (Figure 5). The better release of the drug from all emulgel formulations can be observed, and the emulgel formulations can be ranked in the following descending order: F5 > F3 > F4 > F1 > F2 > F7 > F6 > where the amounts of the drug released after 12 h were 56.78, 61.3, 77.41, 84.41, 85.52, 91.86, and 93.31%, respectively. The best drug release was observed with formulations F3 and F5. This finding may be due to the presence of liquid paraffin at its low level and the gelling agent at its high level, respectively [37,38].

### 2.5. Scanning Electronic Microscopy (SEM) *[39,40]*

**Figure 6:**
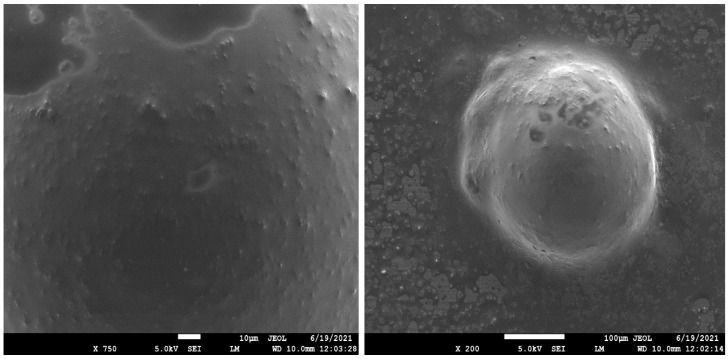
Scanning electron microscopy of liposomal emulgel with Eucalyptus oil and clove oil.

The SEM studies revealed that the shape and size of the liposomes were smooth and spherical (Figure 6). These results proved that the spherical shape of vesicles was unaggregated and unilamellar with a smaller size so that there would be uniformity in the release of drug [41].

### 2.6. Transmission Electron Microscopy (TEM) *[40,41]*

**Figure 7:**
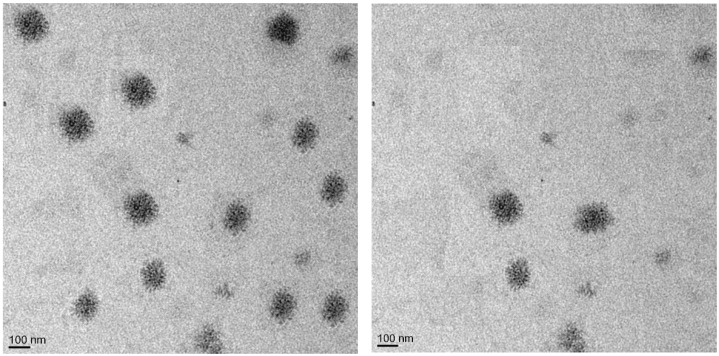
Transmission electron microscopy of liposomal emulgel with eucalyptus oil and clove oil.

The TEM studies revealed that the dispersion of liposomes was spherical in shape and non-aggregated (Figure 7). These results confirmed that the liposomal emulgel had uniform vesicular size with minimum variation, thereby minimizing the fluctuation in dose and release profile. The smaller and unilamellar size of the liposomal emulgel could be due to the presence of oils in these formulations [42].

### 2.7. Cytotoxicity Study: *[43,44]*

**Figure 8:**
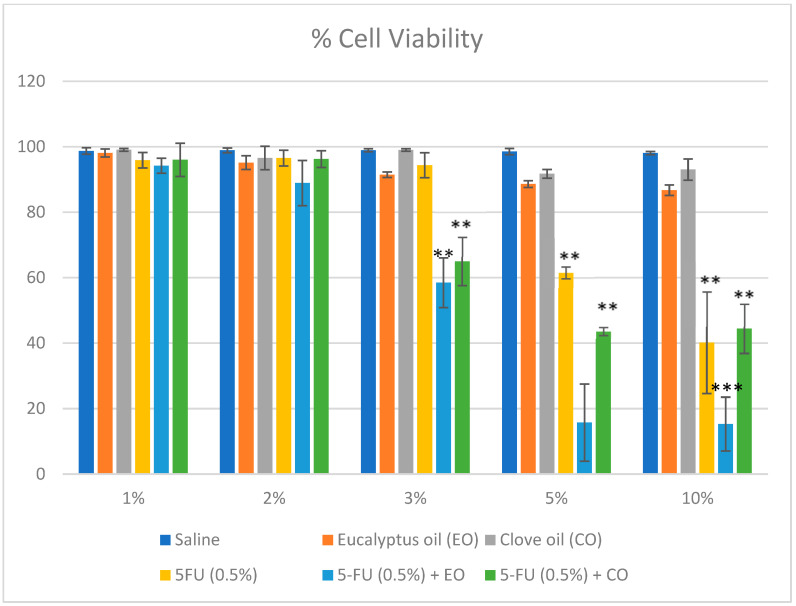
Percentage cell viability of B16-F10 cells against various treatment groups. The values were statistically analyzed by Student’s *t*-test (*n* = 3). Values were compared with saline-treated groups. ** *p* < 0.01, *** *p* < 0.001.

The MTT assay was used for the analysis of a 5% 5FU formulation in a plain liposomal preparation and a preparation with 5% eucalyptus oil and clove oil on a mouse skin melanoma cell line (B16-F10). The cell-killing strength of formulations was determined at 1 mg/mL. As demonstrated in Figure 8 the liposomal 5% eucalyptus oil and clove oil-containing preparation significantly depleted the count of viable cells to 15.74 ± 11.78% compared to plain 5FU liposomal emulgel (40.15 ± 15.54%). In another way of interpretation, the liposomal 5% eucalyptus oil and clove oil-containing preparation significantly killed melanoma cells at 84.26%, compared to the plain 5FU liposomal emulgel (59.85%). Obviously, there was no significant effect on cell viability by eucalyptus and clove oil at 1–3% concentration. However, at 5% concentration, it has a significant effect (*p* < 0.05) on the cell viability. The potential effects (*p* < 0.001) observed were when 5FU was combined with 5% eucalyptus and clove oil. Comparatively, eucalyptus oil had only 15.74 ± 11.78, compared to 43.61 ± 1.23 of the same strength in clove oil and 61.53 1.8% in plain 5FU. These results agree with the results of eucalyptus oil in T24 human bladder epithelial cells [45] and clove oil in human skin cells [46]. The cell viability assay of the selected cancer cell lines indicated that it successfully decreased cell viability at a 10x lower dose with higher efficacy compared to a plain liposomal preparation.

Squamous cell carcinoma is one of the most common forms of non-melanoma skin cancer and accounts for 20% of all cases. The chemotherapeutic treatment with 5FU is difficult because of the very hydrophilic nature of the compound, which results in limited penetration and compromised efficacy. A recent study [47] recommends the use of 5FU liposomes with penetration enhancers such as sorbtitan monolaurate and span 20 in order to improve the drug’s efficacy and its ability to reach its target. Based on the findings of the present investigation, we assume it would be worthwhile to prepare a liposomal emulgel of 5FU with eucalyptus and/or clove oil and test its efficacy against squamous cell carcinoma cell lines.

In the development of an emulgel formulation, it is necessary to exercise extreme caution while selecting the agents that will emulsify and gel the mixture. The use of effective emulsifying agents in topical drug delivery systems helps to achieve a more favorable interaction with the water and lipids found in the stratum corneum. Because of their more biocompatible physico-chemical features, O/W emulsions are typically preferred over other types. The incorporation of gelling agents makes it simple to transform these emulsions into emulgels in the desired form. Emulgels are a fairly well-established type of pharmaceutical topical formulation due to their superior stability, controlled drug release, soothing effect, non-irritancy, simplicity of application, and improved spreadability [48]. In the course of this research, we paid great attention to selecting emulsifying and gelling agents, as well as surfactants and co-surfactants, all of which contribute to the formulation’s stability and enhanced topical penetration. Topical drug delivery was preferred in this case because it bypasses metabolism, has a minimum pH variation, minimizes enzyme interference, reduces empty stomach time, and causes less discomfort; hence, the majority of researchers, academicians, and research scholars are focusing their efforts on developing topical drug delivery systems [49].

## 3. Conclusions

This research was done to develop a liposomal emulgel of 5FU with increased skin permeability and efficacy using clove oil and eucalyptus oil for skin cancer at a low dose. The 5FU formulations were subjected to various evaluation parameters, such as percentage yield, percentage drug content, and in vitro drug release. The physical appearance and melting point of the drug were found to be concordant with those mentioned in USP (2002), which show the purity of the sample. The solubility of 5FU was found to be soluble in methanol, sparingly soluble in ethanol, slightly soluble in PBS (pH 5.5), and insoluble in water. Instrumental characterization supported the stability of the formulation; its increased duration of release, improved bioavailability, and dissolution rate were due to changes in the crystalline phase of 5-fluorouracil on application of stress. The drug and excipient compatibility was confirmed by performing FTIR and DSC investigations. The SEM and TEM studies revealed that the size and shape of the liposomes were smooth and spherical, and the liposomes were non-aggregated with each other. SEM analysis also revealed that the drug molecules were well and uniformly distributed throughout the formulation. It also showed that the 5FU, HPMC, and excipients were consistent and well blended in each formulation. TEM results indicated that 5FU was embedded within the colloidal fat, especially when prepared, and had better uniformity with respect to particle size and shape, entrapment of 5-fluorouracil, and dispersion throughout the emulgel. The eucalyptus oil and clove oil-containing preparation significantly killed melanoma cells compared to plain 5FU liposomal emulgel.

## 4. Materials and Methods

### 4.1. Drugs and Chemicals

FU was received as a gift sample from Sun Pharma, Mumbai, India, and cholesterol and phospholipids extracted from Sorbitol/Mannitol were purchased from CDH Laboratory, New Delhi. All other chemicals and reagents were purchased from Merck Ltd., Bengaluru, India. Clove oil purchased from local market was obtained by cold compression of *Syzygium aromaticum* belongs to family *Myrtaceae*. Eucalyptus oil purchased from local market was obtained by cold compression of *Eucalyptus tereticornis* belongs to family *Myrtaceae*. All the chemicals used in the study are of AR grade.

### 4.2. Drug-Excipient Compatibility Studies

Drug interaction links were studied using FTIR recognition. The FTIR display was cut from the standard KBr disc method using the Shimadzu FT-IR spectrophotometer.

### 4.3. Fourier Transform Infrared Spectroscopy (FTIR) *[50]*

With the help of a Fourier transform infrared spectrophotometer with wave numbers ranging from 4000 to 400 cm^−1^ [51], infrared spectral analysis of the pure drug 5FU was performed and then compared to the spectral analysis of 5FU formulated emulgel. The intensity was measured as the percent transmittance of IR radiation. IR spectroscopy was performed using the FTIR-8400S spectrophotometer (Shimadzu, Japan). The completely dried drug (5mg) was mixed with potassium bromide (KBr) individually. The powder was pressed to make its pellets using a KBr press for 15 tons [52].

### 4.4. Diffraction and Scattering Techniques (DSC)

Differential scanning calorimetry (DSC) and thermogravimetric analyses (TGA) were carried out on solvent-cast 5FU-loaded and blank PU films and on pure 5FU drug using a Discovery DSC 2920 (TA Instruments, New Castle, DE, USA) and a Discovery TGA 550 (TA Instruments), respectively. For DSC, accurately weighed films, or 5FU (~2 mg), were placed in aluminum pans and hermetically sealed, and then measurements were performed at a heating rate of 10 °C/min between 20 and 600 °C under a nitrogen atmosphere. For TGA, ~7.6 mg of sample was placed in a platinum pan and then heated under a nitrogen gas flow at a rate of 10 °C/min up to 500 °C [53].

### 4.5. Thin Film Hydration Method for the Preparation of Liposomes

**Figure 9:**
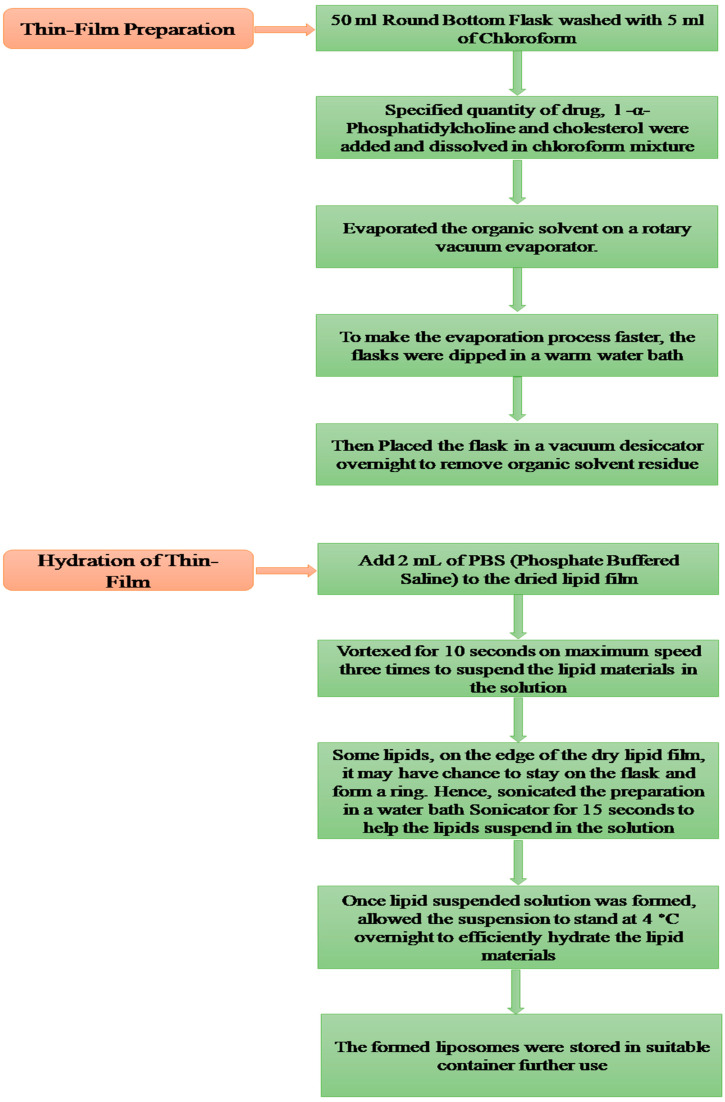
Schematic presentation of the method of preparation of the formulation.

Thin Film Hydration Method for the Preparation of Liposomes (Figure 9). 

### 4.6. Preparation of 5FU Liposomal Emulgel

Emulgel was prepared by adding hydroxypropyl methylcellulose (HPMC), a gelling agent, to sterile water and stirring the mixture at a slow to medium pace. The pH was adjusted using triethanolamine (TEA), the oil phase was prepared by dissolving span 80 in light liquid paraffin, and the aqueous phase was made by dissolving tween 20 in purified water (Table 1). In one beaker, sodium benzoate was diluted with propylene glycol. The drug was combined with ethanol in a separate beaker, and then all three phases were blended. The oil and water were heated to temperatures between 70 and 80 degrees Celsius on their own. The emulgel was made by combining the oil and water phases with constant stirring until room temperature was reached, at which point the eucalyptus and clove oils were added at a ratio of 1:1 throughout the mixing process (Figure 9).

### 4.7. Post-Formulation Studies

#### 4.7.1. In-Vitro Drug Release Study [54]

For drug release studies, the Franz diffusion cell with an effective diffusion area of 3.14 cm^2^ and a cell volume of 13 mL was used. An egg membrane, which is commonly known as the chicken embryo chorioallantoic membrane (CAM) model, was selected for this study based on the fact that it is affordable, time-efficient, appropriate for across biological membrane diffusion studies, and has similarity to the cell membrane [55]. On the surface of the egg membrane, 200 mg of emulgel were applied. After this, clamping of the egg membrane between both chambers (i.e., the donor and receptor) of the diffusion cell is done. For solubilizing the drug, freshly prepared PBS with a pH of 7.4 was filled in the receptor chamber. With the help of a magnetic stirrer, the receptor chamber was stirred continuously. At a specific time, an interval sample was collected and examined for drug content after preparing different dilutions by using a UV-visible spectrophotometer. For getting the total amount of drug release onto each time interval, cumulative corrections were made.

#### 4.7.2. Negative Stain Transmission Electron Microscopy (TEM) [56]

Staining with a 2% phosphotungstic acid solution was performed after placing 10 μL of the liposomal formulation on carbon-coated copper grids and drying it under a lamp. Afterwards, Milli-Q water was used to clean it. After drying, the sample was placed into the receiver, and a vacuum was generated so TEM pictures could be taken. Using SIS software, the diameter of the particles was determined at various magnification levels.

#### 4.7.3. Scanning Electron Microscopy (SEM)

To create topographical pictures of the abluminal (outside) surfaces of the samples, platinum was sputtered (thickness ~5 nm) using an automatic sputter coater and applied to sections of coated stents, which were subsequently placed horizontally on double-sided sticky carbon tape fastened on an aluminum SEM stub. With an accelerating voltage of 2 kV, images were taken with a Zeiss Merlin field emission gun (FEG) scanning electron microscope. The stent segments were placed vertically on a custom-made SEM stub and fastened with a screw to facilitate cross-sectional imaging.

#### 4.7.4. Cytotoxicity Study [42,43]

The current innovation was studied by the in vitro method on the cell line B16-F10 of mouse skin melanoma, purchased from the National Centre for Cell Science (NCCS), Pune. The (3-(4,5-dimethylthiazol-2-yl)-2,5 diphenyl tetrazolium bromide) (MTT) assay is an in vitro colorimetric assay employed for the quantitative cytotoxic efficacy of developed formulations. The principle of the assay is based on the conversion of tetrazolium salt (yellow) to formazan, an insoluble crystal (purple), by the mitochondrial enzyme succinate dehydrogenase present in the viable cells. The MTT reagents come across the cells and pass into the mitochondria, where the tetrazolium salt (yellow) gets reduced to an insoluble, dark-purple formazan product. The culture cells were treated with a liposomal emulgel prepared with 5% eucalyptus oil and 5FU, the liposomal emulgel containing only 5% FU, and the released formazan reagent (solubilized) was estimated spectrophotometrically. MTT biologically acts on metabolically active cells, and the level of biological activity of the cells indicates cell viability. Cell lines were seeded on a 96-well plate (10^6^ cells/well) containing DMEM media with 4 mM L-glutamine adjusted to contain 1.5 g/L sodium bicarbonate, 4.5 g/L glucose, and 10% fetal bovine serum, and then incubated overnight at 37 °C in a humidified atmosphere air enriched with 5% (v/v) CO_2_ in order to attach the cells to the bottom of each well. The cultured cells were then treated with different concentrations to determine the cytotoxicity of formulations after 48 h of incubation.

After treatment, the medium was carefully removed and further incubated with 10 µL of MTT in a fresh medium under a CO_2_ incubator for 3 h. After an incubation period, the insoluble crystals (Formazan) were dissolved in the DMSO solubilizing agent (130 μL/well), and absorbance was measured at 570 nm in a microplate reader (Microlisa Plus, India). Each experiment was performed in triplicate (*n* = 3). Saline-treated cells were considered the control, with 100% cell viability. Cell viability data were calculated as per the previous study. The cell viability (%) was represented as mean viability (%) ± standard deviation (SD) (*n* = 3), and the results were statistically analyzed by Student’s *t*-test to assess the level of significance. *p* < 0.05 was considered significant. The percent cell viability and cell death were calculated using the formula as shown below:

Percent cell viability = OD treated/OD controlled × 100

Percent cell death = 100 − Cells Viable

## Figures and Tables

**Table 1:** The formulations F1–F7 design and composition.

Ingredients	*%w/w*						
	F1	F2	F3	F4	F5	F6	F7
5-fluorouracil	0.5	0.5	0.5	0.5	0.5	0.5	0.5
HPMC	0.05	2.0	0.05	2.0	0.05	0.2	0.05
Triethanolamine	0.02	0.02	0.02	0.02	0.02	0.02	0.02
Light liquid paraffin	3.5	2.65	3.5	2.65	3.5	2.65	3.5
Tween 20	0.5	0.3	0.3	0.3	0.5	0.5	0.5
Propylene glycol	3.5	3.5	3.5	3.5	3.5	3.5	3.5
Sodium benzoate	0.5	0.5	0.5	0.5	0.5	0.5	0.5
Ethanol	1.55	1.55	1.55	1.55	1.55	1.55	1.55
Eucalyptus oil	1	5	-	-	-	5	1
Clove oil	-	-	1	5	-	1	5
Purified water (q.s)	50	50	50	50	50	50	50

## Data Availability

Data is available with first author of the article.
